# On Amalgam

**Published:** 1870-11

**Authors:** James Lewis


### ARTICLE V.

### *On Amalgam.*

By Dr. James Lewis.

From my earliest recollection, the Eclectic school of medicine, (as the botanists delight to be called,) have denounced mercury as an unsafe, aye, as a deleterious medicine.—Homœopathy, also, contribute its quota to the same denunciation; and though they have repeatedly demonstrated their hypotheses to their own satisfaction, mercury, in the form familiarly known to the physicians as submuriate, (Hy. Cl.,) continues to be the *sheet-anchor* of medical practice.

From my earliest acquaintance with dentistry I have been perplexed with the discussions relating to amalgam. Perhaps I have been unfortunate in never having found any peculiar conditions of the mouth that could, by the most remote possibility, have been attributed to the influence of mercury derived from an amalgam plug. I have examined a great many teeth that have been filled with this material, and when I have found the parts around them diseased, the origin of the difficulty was so entirely referable to conditions in which mercury was not involved, that to intimate mercurial disease would be unpardonable presumption. There are, undoubtedly, instances in which, on account of a peculiar diathesis, a patient is intolerant of mercury in any of its forms, (except as administered homœopathically—in which case a mathematical "differential" may be placed as the coefficient of the *quantity used.*) I have never, in dental practice, seen a patient possessing the peculiar diathesis. I will admit, however, that for such a person an amalgam plug, under certain conditions, *might* be a dangerous thing. We find persons who are also intolerant of opium to those persons who have no such idiosyncrasies; and when we have a patient who will bear calomel, its use, in given cases, is to be recommended, not condemned. To go back for another illustration: If we deprive ourselves of a useful agent, because the ratio of its *dangers* to its *utility* may be as 1 to

100, or even more, we might, with propriety, insist upon the abandonment of ocean steam navigation, for the reason that, occasionally, a vessel is so irrevocably lost at sea that the "spirit mediums" fail to render an accout of the fate of its passengers.

Much of the argument against amalgam and its uses is simply *specious*. Writers who delighted to ventilate their ideas on this subject unconsciously pervert many of the most important facts and principles involved in our knowledge of electro-metallurgy, and their *speculations* upon the probable manner in which electric or electro-chemical forces manifest themselves in the human mouth, in connection with the substance of the teeth and of amalgam, show, to one who has patiently studied the laws of electro-chemical force, that they, (the writers,) have not yet reached even a tolerable comprehension of the subject; and when we come to consider their ideas of the pathology of the subject, we shall find, on careful scrutiny, that in some cases there is a palpable error of diagnosis, in others a disposition to condemn a substance, which, being injurious in one case, may be the *best* that is available in ninety-nine others.

I do not *advocate* the use of amalgams. I am just enough conservative not to deprive myself of a useful agent on account of a *prejudice*. At one time I was considerably influenced by the appeals of writers who sought to create a *prejudice* in the minds of the profession—(the writers themselves being evidently prejudiced)—and, for a considerable time, I argued with my patients to induce them to forego their use. It is scarcely necessary to say that this mode of doing business did not prove satisfactory, and I was *obliged* to use amalgam. Since that period I have studied amalgams with much interest, and in this connection I have also considered them as agents for the development of electro-chemical force, and their possible relations to the teeth, and to plugs of other material in the same or contiguous teeth. Probably some of my readers know the *secret* of the *white* amalgams. The best of them have the silver alloyed with

*cadmium* or *zinc*. For certain electro chemical reasons, zinc is preferable. The part that zinc plays in the compound is to protect the silver and mercury from the oxidizing influences of the fluids of the mouth. Zinc being the most highly electro-positive of any of the metals that can be used in such preparations, commends itself on the electro-chemical theory as an important adjunct in amalgam compounds.

Perhaps it may not be out of place to give my mode of preparing amalgam compounds. This I will endeavor to do as succinctly as possible, with my *reasons* for each particular. I prefer *coin silver* alloyed with *copper*. The presence of *copper* insures a greater degree of *hardness* than is attainable when it is absent, and in the presence of zinc it is as fully protected from electro-chemical action as the silver or mercury is. I melt the silver, and if it be chemically pure I add copper, about 8 per cent. to the argento-cupreous alloy. I add, while in fusion, zinc, about 5 per cent., after which the metal is cooled as quickly as possible. The alloy is then reduced (by means of suitable cutters secured on a mandrel in a lathe) to fine cuttings, which are afterwards passed through a very fine screen or sieve of brass wire gauze. To this I add also about 5 per cent. of *similarly screened cuttings* of *metallic zinc*, which helps to impart the necessary electro-positive character to the mass. Finally, I add from 50 to 80 per cent. *chemically pure silver* in *atomic division*, regulating the amount empirically, as it seems to be needed on testing portions of the mass from time to time.

I prepare my chemically pure silver by dissolving refuse silver, scraps and old plugs of amalgam in nitric acid. Filter the solution; add sufficient hydrochloric acid or chloride of sodium to precipitate all the silver as chloride, which chloride should be rapidly and thoroughly washed by frequent changes of pure water. The reason why I use chemically pure silver in atomic divisions is this: The *cuttings* of silver, no matter how fine, will have appreciable interstices between them, and an amalgam prepared from fillings or cuttings alone will inevitably become porous by the process of saturation—the little fragments of silver being at first simply *coated* with mercury, finally absorb more, which necessarily drains the interstices If, in the preparation of an amalgam, we can introduce some substance which will not greatly disturb the homogeneousness of the mass, and which will displace as much mercury as possible, we shall have secured two advantages. First, we obviate porosity. Second, we have to use *less mercury*. And in the result we obtain a *condition* of the mass in which mercury is obviously not available to the extent observed in some other forms of amalgam, for the production of such salts as may act prejudicially upon the tissues in the vicinity. In such an amalgam as I prepare the mercury cannot be twenty per cent. In a plug weighing five grains, we would find less than one grain of mercury. Allowing as large a surface of exposure as possible, which very seldom would reach as much as 0.25 square inches, the query arises: What fraction of a grain of mercury can possibly be absorbed from this mass in given time? and if it permeates the tissues as a *soluble salt*, how does it reach them? Is it through indigestion after undergoing solution in the mouth by the action of perverted saliva, or does the mercury in a metallic form pervade the structures of the tooth, or does it undergo solution in fluids pervading the tubuli of the teeth, and then find its way slowly into the adjacent tissue? We may declare our *opinions* on these points, but that is *not* demonstration. As I said before, much of the reasoning on these queries is too *specious*, and it seems to me to assume the character of *special pleading* to sustain an *a priori* hypothesis. The chloride is then covered with water, say half an inch deep, sufficient sulphuric acid and metallic zinc being added to insure the reduction of the chloride to pure silver. When this is fully accomplished, as may be known by the entire disappearance of the white chloride, and the flaky or granular appearance of the dark metallic silver, the zinc may be removed, and the silver remain a few hours in contact with the unappropriated sulphuric acid to insure the solution of any remaining undissolved portions of zinc. The silver is finally to be thoroughly washed with clean water, until *clean*, when it may be squeezed dry, or nearly so, in a napkin, after which it may be crumbled with the fingers, and more completely dried on a metallic plate over a lamp.

A few words relating to my mode of using this amalgam may not be out of place. Let the cavity be as carefully prepared as for a first-class gold filling. If the pulp cavity be endangered by the pressure necessary to insure a firm filling, cap with *osteoplastic*, or in any other mode that will secure permanence and security, dry the cavity by removing surplus saliva with a syringe, completing the process with any of the absorbents usually applied for that purpose. But, before the final drying of the cavity, mix the amalgam, and while it yet has a surplus of mercury, wash it with pure waters, to which a few drops of ammonia (alkaline) have been added, until the amalgam is *perfectly clean*. Remove surplus mercury by squeezing in a napkin, or any other convenient, suitable material. The amalgam is now to be packed firmly in the carefully dried cavity, using a surplus, in order to take up and remove the free mercury forced out of the mass by condensation, taking care that this part of the operation is thorougly and efficiently performed. Dress the surface of the plug to a *contour*, and burnish it. After the plug has become somewhat hardened the burnishing may be repeated until the surface remains bright and smooth. Plugs of this character, in an average mouth, retain their brightness and seldom discolor a tooth—the *vitality* of which is sufficient to retain a plug of other material.

In *dead teeth* discoloration is very liable to ensue. This may be explained by attributing it to the evolution of gases containing *sulphur compounds*, which, it is known, are often evolved in appreciable quantities, from the decomposition of organic substances, and more especially those of animal origin. I have omitted to enlarge upon some points in my remarks, where it appears to me facts and reasons are so obvious as to suggest themselves.

In the amalgam question I do not propose to enter at large. Most of the discussions that are likely to arise in considering a subject in which the theory and practice of different persons is as different as can be, argument is so liable to degenerate into controversy, that the *main point* is lost to view in an endeavor to silence an opponent. A writer may sometimes be too busy or *too indolent* to fight his way out of a discussion, from which he will emerge without having achieved anything but opposition. In this matter I think I may claim the privilege of my age to plead an excusable amount of indolence.—*Dental Times.*

